# Putative Mechanisms Underlying High Inhibitory Activities of Bimodular DNA Aptamers to Thrombin

**DOI:** 10.3390/biom9020041

**Published:** 2019-01-24

**Authors:** Elena G. Zavyalova, Valeriia A. Legatova, Rugiya Sh. Alieva, Arthur O. Zalevsky, Vadim N. Tashlitsky, Alexander M. Arutyunyan, Alexey M. Kopylov

**Affiliations:** 1 Chemistry Department, Lomonosov Moscow State University, 119991 Moscow, Russia; lera000006@gmail.com (V.A.L.); ruqiwa_eva@mail.ru (R.S.A.); tashlitsky@belozersky.msu.ru (V.N.T.); kopylov.alex@gmail.com (A.M.K.); 2 Faculty of Bioengineering and Bioinformatics, Lomonosov Moscow State University, 119234 Moscow, Russia; aozalevsky@gmail.com; 3 Belozersky Research Institute of Physico-Chemical Biology, Lomonosov Moscow State University, 119991 Moscow, Russia; alarut@genebee.msu.ru

**Keywords:** DNA aptamer, G-quadruplex, conformational polymorphism, thermodynamics, thrombin, structure–activity relationship

## Abstract

Nucleic acid aptamers are prospective molecular recognizing elements. Similar to antibodies, aptamers are capable of providing specific recognition due to their spatial structure. However, the apparent simplicity of oligonucleotide folding is often elusive, as there is a balance between several conformations and, in some cases, oligomeric structures. This research is focused on establishing a thermodynamic background and the conformational heterogeneity of aptamers taking a series of thrombin DNA aptamers having G-quadruplex and duplex modules as an example. A series of aptamers with similar modular structures was characterized with spectroscopic and chromatographic techniques, providing examples of the conformational homogeneity of aptamers with high inhibitory activity, as well as a mixture of monomeric and oligomeric species for aptamers with low inhibitory activity. Thermodynamic parameters for aptamer unfolding were calculated, and their correlation with aptamer functional activity was found. Detailed analysis of thrombin complexes with G-quadruplex aptamers bound to exosite I revealed the similarity of the interfaces of aptamers with drastically different affinities to thrombin. It could be suggested that there are some events during complex formation that have a larger impact on the affinity than the states of initial and final macromolecules. Possible mechanisms of the complex formation and a role of the duplex module in the association process are discussed.

## 1. Introduction

Nucleic acid aptamers are prospective molecular recognizing elements with a high potential in both recognition studying and applied research for diagnostic and therapeutic applications. Aptamers attract attention due to high affinity, specificity, and exceptional biocompatibility [1]. One of the most elaborated areas of aptamer studying is in the inhibition of blood clotting, in particular blocking thrombin function [2,3,4,5]. Several promising candidates were found to provide anticoagulant therapeutic effect in vivo during preclinical and clinical trials [2,3,6].

Aptamers are considered as chemical analogues of antibodies, as specific recognition is provided by certain spatial 3D structures of the aptamer. Thousands of aptamers have been described to date, and structures of dozens of aptamer–protein complexes have been resolved; however, reasons of the extremely high affinity of some aptamers are nebulous. Despite particular examples of a clear structure–activity relationship [7], in general, no clear way has been found to improve the affinity of the specific aptamer [8]. Thus, the main route of aptamer creation and improvement is still a selection from a huge combinatorial library. Here, we focus on searching for the main features that are responsible for the high affinity of aptamers by studying a set of bimodular thrombin aptamers as an example.

**Figure 1:**
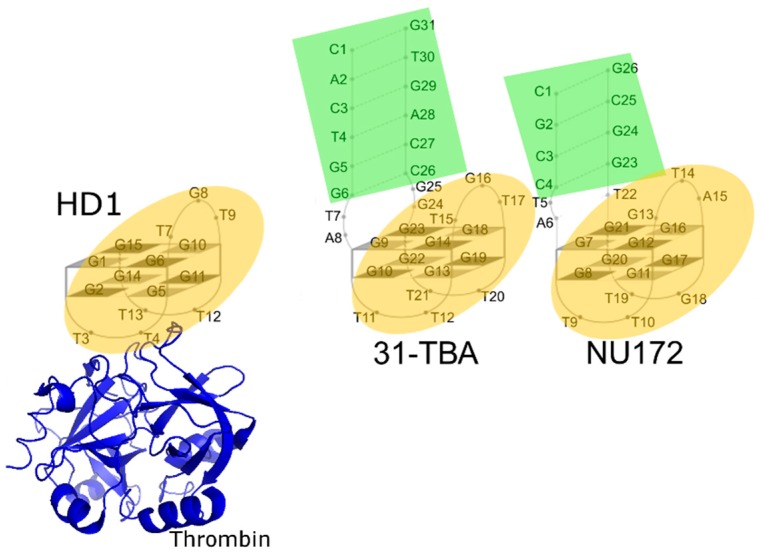
Schematic drawing of complexes of aptamer HD1 with thrombin and HD1-like aptamers 31-TBA and NU172. Modules are indicated by color: duplexes in green and G-quadruplexes in orange. Aptamer HD1 recognizes and binds thrombin exosite I with TT-loops (T_3_T_4_ and T_12_T_13_).

Most of the DNA aptamers to thrombin have the structure of antiparallel chair-like G-quadruplexes, the two bottom loops (like ‘legs’) of which are responsible for thrombin binding. Aptamer HD1 represents the minimal core structure sufficient for thrombin binding with high affinity and specificity (Figure 1). The ultimate requirement for the HD1 binding to thrombin is T_4_T_13_-pair that forms a set of hydrogen bonds with thrombin, whereas two other thymines from the ‘legs’ (T_3_ and T_12_) can be altered. The same requirement is fair for all HD1-like aptamers that bind thrombin exosite I. Additional structural elements/modules could enhance the affinity and affect the selectivity for the thrombin precursor, prothrombin, whereas their mode of inhibitory action on thrombin is the same [9,10,11,12]. As for affinity improvement, bimodular aptamers, 31-TBA, NU172 (Figure 1), and RE-31, are made up of the duplex module added to the core G-quadruplex structure. They have apparent inhibitory/dissociation constants as low as 0.3–0.5 nM, which is 30 times lower than that of HD1 [9,12]. Aiming to study phenomenon of increased affinity, a set of hybrid derivatives was created by exchanging the modules between 31-TBA and NU172. The resulting aptamers had the same mode of inhibitory action on thrombin and a wide range of apparent inhibitory constants, 1–50 nM [9]. Therefore, studying the structures of this set of aptamers could reveal some determinants of the high affinity of the aptamers and provide some understanding of the structure–activity relationship.

The apparent simplicity of oligonucleotide folding into a proper spatial structure for the aptamer is often elusive; a balance between several conformations is controlled by a number of factors, like temperature, buffer composition, including the nature of cations, oligonucleotide concentration, etc. [13,14,15]. Here, we demonstrate a conformational polymorphism for some low-affinity bimodular aptamers and the strict homogeneity of high-affinity aptamers by using spectroscopic and chromatographic techniques. Known structures of HD1-like aptamer–thrombin complexes were thoroughly compared in terms of interface characteristics and thermodynamic properties of the aptamers. A tentative mechanism of aptamer binding to thrombin has been proposed.

## 2. Materials and Methods

Oligonucleotides were chemically synthesized by Evrogen (Moscow, Russian Federation). The sequences are provided in Supplementary Table S1. Salts for buffers were of analytical grade purity from MP Biomedicals (Illkirch, France). O’GeneRuler Ultra Low Range DNA Ladder was purchased from ThermoFisher Scientific (Vilnius, Lithuania).

Oligonucleotide samples were prepared in 2 µM concentration in 20 mM Tris-HCl buffer, at pH 7.4, with 140 mM NaCl, 5 mM KCl, 1 mM MgCl_2_, and 1 mM CaCl_2_. The samples were heated at 95 °C for 5 min, and then cooled to room temperature. The concentrations of cations correspond to the physiological conditions of blood plasma; this buffer is the same as used for functional tests earlier [9].

### 2.1. Size-Exclusion Chromatography

Size-exclusion chromatography (SEC) was conducted with an Agilent 1200 HPLC system with autosampler and diode array detector (Agilent; Santa Clara, CA, USA). The HPLC column TSKgel G2000SWXL (Tosoh Bioscience; King of Prussia, PA, USA) had the following parameters: 30 cm length, 0.78 cm diameter, 5 µm diameter of particles, 12.5 nm mean pore diameter. The column is intended for the separation of proteins with molecular weights in the range 5–150 kDa. The separation conditions were the following: temperature 25 °C, mobile phase water/acetonitrile 9:1 v/v with potassium phosphate buffer (60 mM KH_2_PO_4_ and 140 mM K_2_HPO_4_, pH 6.85), flow rate 0.5 mL/min. Absorption at 260 nm was registered with 10 nm bandwidth. The DNA ladder was purified from low molecular weight dyes by using VivaSpin 500 columns with polyethersulfone (PES) membrane and pores suitable for elution of compounds below 3 kDa (Sartorius; Epsom, UK). Analysis of aptamer samples did not require additional manipulations.

### 2.2. Circular Dichroism and UV Melting

Circular dichroism (CD) spectra were measured by using the CD spectrometer CHIRASCAN (Applied Photophysics Ltd.; Leatherhead, UK) and dichrograph MARK-5 (Jobin-Yvon; Montpellier, France) equipped with a thermoelectric temperature regulator. Quartz cuvettes with optical path length of 1 cm were used. CD and UV spectra were obtained in the wavelength range 220–350 nm and temperatures range 7–70 °C with an average heating ramp of 0.5 °C/min. Five samples with two isodichroic points in CD spectra were cooled with the same ramp to check hysteresis. Buffer was used as a blank sample and its spectrum was automatically subtracted from the oligonucleotide spectra. The CD signal was recalculated as molar circular dichroism, Δε (cm^−1^ M^−1^).

All data treatment and calculations were made by using OriginPro 8.0 software (Origin; Northampton, MA, USA). Melting temperatures were calculated by using the Boltzmann approximation of melting curves at a certain wavelength. Thermodynamic parameters were calculated by assuming two-state equilibrium [16].

### 2.3. Analysis of Structures of Aptamer–Thrombin Complexes

Structural analysis of aptamer–thrombin complexes was performed by using PyMol software, version 1.74 (http://pymol.org). Derived parameters included lengths and angles of contacts, a number of atoms in the interface, and a number of polar contacts.

## 3. Results

### 3.1. Testing Oligomeric Composition with Size-Exclusion Chromatography

**Figure 2:**
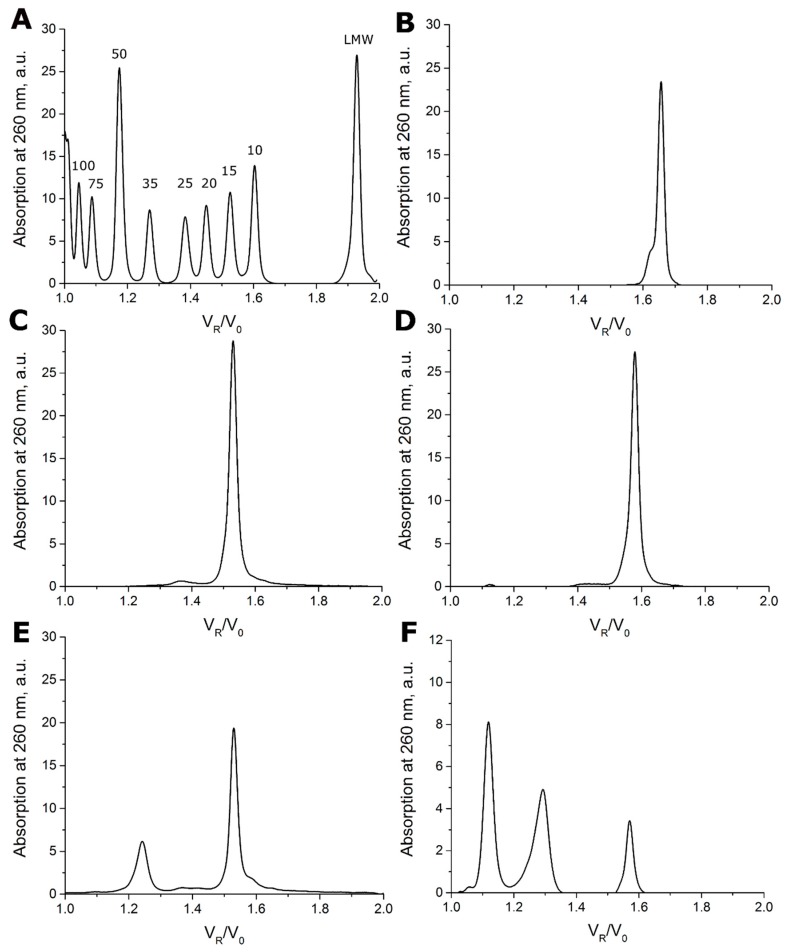
Chromatograms of size exclusion HPLC for the DNA ladder (**A**), aptamers HD1 (**B**), 31-TBA (**C**), NU172 (**D**), jja (**E**), and ajj (**F**). V_R_/V_0_ is the relative retention volume. Lengths of DNA duplexes from the DNA ladder are indicated, LMW refers to low molecular weight substances, most likely, residual amounts of dyes from the ladder.

G-blocks in the aptamer pattern could participate both in unimolecular and intermolecular G-quadruplex folding. For the latter case, the different oligomeric composition is one of obvious reasons that can explain the broad variations in the inhibitory constants of the bimodular aptamer family, 1–50 nM [9]; constants are given in Supplementary Table S1. The set of aptamers was analyzed by size-exclusion chromatography (SEC). The examples of chromatograms of the DNA ladder, HD1 and several bimodular aptamers are shown in Figure 2; all other chromatograms are provided in Supplementary Figure S1. Control DNA duplexes in the range from 10 to 100 base pairs were readily separated with SEC (Figure 2A). Calibration of the column (Supplementary Figure S1B) allows direct determining of the molecularity of the aptamers. The data are summarized in Table 1. Original aptamers—HD1, 31-TBA, and NU172—were strictly monomeric (Figure 2B–D). Half of the hybrid bimodular aptamers, aaj, jaa, and jaj, retain monomolecularity; whereas aptamers jja, aja, and ajj contained oligomeric fractions (Table 1).

The calculated molecularity values for the oligomeric forms of jja, aja, and ajj with V_R_/V_0_ = 1.24–1.29 were 2.81, 2.65, and 2.67, respectively. These values most likely correspond to the dimeric forms; 1.3–1.4-fold overestimated molecularity values could be a result of the presence of unfolded regions in the dimers, which increase apparent volume of the molecule. To elucidate this phenomenon, highly ordered aptamer HD1, unstructured oligonucleotide complementary to HD1 and the duplex made with these oligonucleotides were analyzed with SEC (Supplementary Table S2). The complementary unstructured oligonucleotide had a 1.46-fold overestimated molecularity value, whereas HD1 and the duplex had low deviations from the expected value (less than 20%). Therefore, the molecularities of 2.65–2.81 are likely to correspond to dimeric forms with partially unfolded regions. Similarly, peaks at V_R_/V_0_ = 1.12 correspond to tetrameric forms with 1.3-fold overestimated molecularity.

### 3.2. Studying the Conformational Homogeneity of Aptamers and Stability of Their Modules with Circular Dichroism and UV Spectroscopies

**Figure 3:**
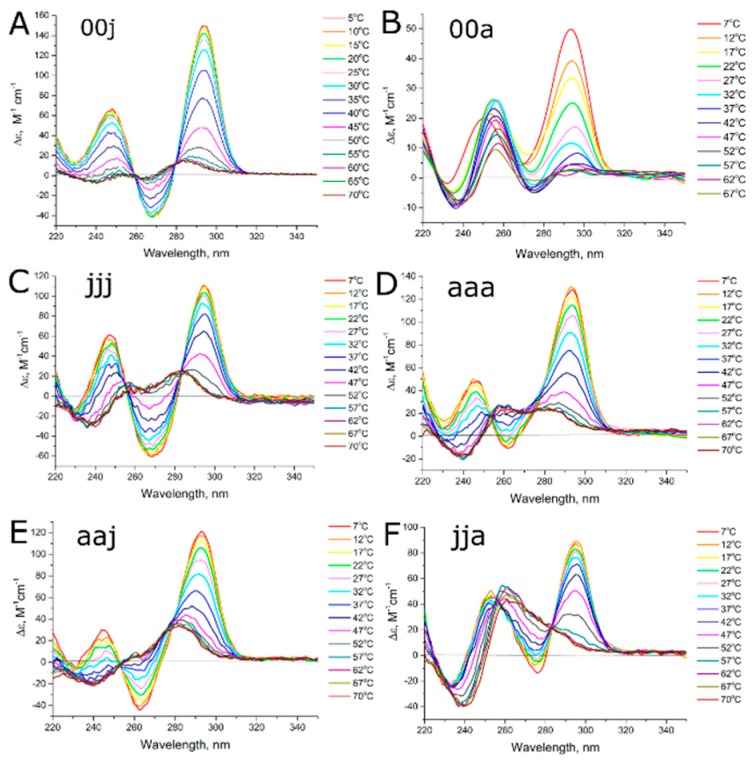
CD spectra of the thermal melting of aptamers (**A**) HD1 (00j), (**B**) NU (00a), (**C**) 31-TBA (jjj), (**D**) NU172 (aaa), as well as hybrid aptamers (**E**) aaj and (**F**) jja.

**Figure 4:**
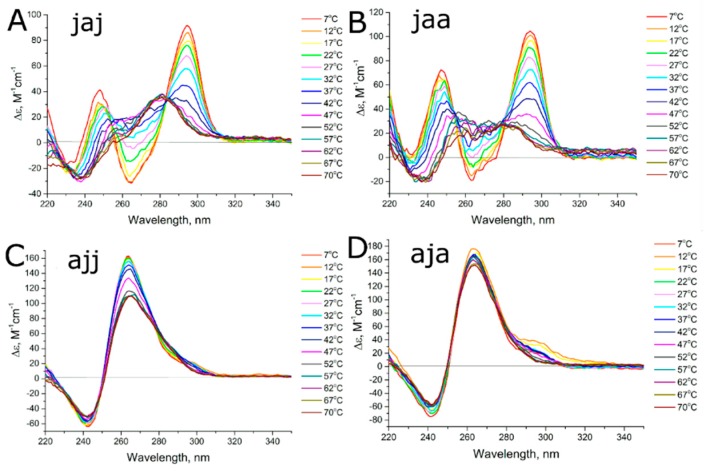
CD spectra of thermal melting of hybrid aptamers (**A**) jaj, (**B**) jaa, (**C**) ajj, and (**D**) aja.

Circular dichroism spectra of a series of bimodular aptamers at different temperatures are shown in Figure 3 and Figure 4. Spectra of G-quadruplex modules alone are provided in Figure 3A,B. Antiparallel G-quadruplexes have characteristic CD spectra with positive maxima at 247 nm and 294 nm, as well as a negative maximum at 267 nm, whereas parallel G-quadruplexes have a positive maximum at 260 nm and negative maximum at 240 nm [17].

At low temperatures, aptamers 31-TBA, NU172, jja, jaa, aaj, and jaj as well as their G-quadruplex modules, HD1 and NU, have the topology of antiparallel G-quadruplexes (Figure 3 and Figure 4). Negative maximum is diminished in case of NU172, jja and jaa. Notably, spectra of unfolded forms of these aptamers contain positive peak at 260 nm, contrary to 31-TBA and HD1; thus, diminished maximum could be a result of superposition rather than altered G-quadruplex topology. Aptamers ajj and aja have the topology of parallel G-quadruplexes (Figure 4), and both these aptamers are predominantly in dimeric and tetrameric forms according to SEC.

Melting experiments were used to study the thermal stability of the G-quadruplex and duplex modules. It was shown previously that CD melting curves (at 295 nm) characterize unfolding of the G-quadruplex module of 31-TBA, whereas UV melting curves characterize both G-quadruplex (295 nm) and duplex modules (260 nm) [18]. The unfolding processes of the two modules of bimodular aptamers were studied with CD and UV spectroscopies. Detailed data are provided in Supplementary Tables S3 and S4, several selected melting curves and their fittings are provided in Supplementary Figures S2 and S3. The results of studying of bimodular aptamers with spectroscopic techniques are summarized in Table 2.

The effect of the duplex module on G-quadruplex stability is demonstrated clearly in Figure 3A–D. Aptamers 31-TBA (jjj), its G-quadruplex module, HD1 (00j) and NU172 (aaa) have two isodichroic points in their melting curves, which indicates a two-state process; melting was assigned to a ‘folded’ to ‘unfolded’ transition. On the contrary, the G-quadruplex module of aptamer NU172 (coined as NU, 00a) has no isodichroic points, exhibiting a repertoire of different conformations. The aptamer has the lowest melting temperature, 17 °C versus 37 °C for bimodular NU172. Therefore, the duplex module of NU172 strongly shifted the equilibrium towards the antiparallel monomolecular G-quadruplex conformation, and hence, prevented conformational diversity.

Only two of the hybrid bimodular aptamers, jja and aaj, have two isodichroic points in their melting curves, that is, they show a two-state melting process (Figure 3E,F). In both cases, the duplex module and hinge loops from one aptamer were placed onto the G-quadruplex module of another aptamer. Interestingly, the aptamer jja had a transition between the two topologies of the G-quadruplex, namely, from ‘antiparallel’ to ‘parallel’, which is unusual for HD1-like aptamers.

Melting curves of aptamers jaa and jaj have only one isodichroic point, which characterizes a transition of antiparallel G-quadruplex to a mix of some other G-quadruplex conformation (probably with parallel topology) and unfolded state. These data could be used to access the thermal stability of the initial conformation only, but thermodynamic parameters could not be estimated. Aptamers ajj and aja are predominantly parallel G-quadruplexes. Small fractions of antiparallel G-quadruplexes were melted at temperatures 47–49 °C; however, these estimations are approximate.

A correlation between the thermal stability of the G-quadruplex module and inhibitory activity of the aptamer has been found. Bimodular aptamers with inhibitory constants below 1.5 nM have spectra of antiparallel G-quadruplexes with melting temperatures in the range 37–49 °C, whereas low-activity bimodular aptamers with a predominant fraction of antiparallel G-quadruplex have melting temperatures of 32–34 °C, which are lower than the temperature used for inhibitory activity assays. Notably, duplex modules of aptamers with a predominant fraction of antiparallel G-quadruplexes have relatively high thermal stability as their melting temperatures are in the range 51–59 °C. Melting temperatures of duplex modules correspond to the final steps of G-quadruplex unfolding. Thus, unfolding of the modular construct is the sequential process. Large unstructured loop destabilizes duplex; similar observations were published previously [18].

### 3.3. Thermodynamics of Aptamer Unfolding

According to the CD spectra of the thermal melting of bimodular aptamers, four examples were identified as having a single transition of the G-quadruplex: ‘folded’ to ‘unfolded’ in the cases of 31-TBA, NU172, and aaj aptamers or ‘antiparallel G-quadruplex’ to ‘parallel G-quadruplex’ in the case of the jja aptamer. Denaturation-folding experiments revealed these aptamers to have equilibrium melting of G-quadruplex modules, whereas duplex modules had hysteresis (Figures S4 and S5). The datasets for four bimodular aptamers and HD1 were used to calculate the thermodynamic characteristics of G-quadruplex modules (Table 3).

Aptamers 31-TBA (jjj) and HD1 (00j) had similar changes of enthalpy and entropy during the thermal melting of the G-quadruplex modules. Therefore, the duplex module has little effect on the thermodynamic parameters of G-quadruplex module. Similarly, aptamers NU172 (aaa) and jja with the same G-quadruplex module but different duplex modules and hinges had the same ΔH° and ΔS° values. The single aptamer aaj had significantly lower ΔH° and ΔS° values than the others, indicating destabilization of the G-quadruplex module by additional modules.

The relative stability of the G-quadruplex module of bimodular aptamers could be estimated by using melting temperatures derived from CD, to give the following order: jja > jjj (31-TBA) > aaa (NU172) > aaj. As was demonstrated for aptamer RE31, which is homologous to 31-TBA with swapped nucleotides inside the duplex module, the G-quadruplex module connects to the duplex module via stacking mediated by the TGT-loop of the G-quadruplex and hinge loops that connect the two modules [19]. The order of stability correlated well with the number of purines in three nucleotide lateral loop (TGT or GTA, in this case) and hinge loops (TA, GG or TA, T). The most stable, jja, has five purines, jjj (31-TBA) has four purines, aaa (NU172) has three purines, and the least stable, aaj, has only two purines. Thus, stabilization or destabilization effects of duplex modules on the G-quadruplex module are mediated by the nature of the hinge loops between the two modules.

As for the structure–activity relationship, there are no direct rules yet, but the aptamer with the destabilized G-quadruplex had 10-fold lower activity than the other bimodular aptamers. However, the reason for the low activity of HD1 is not obvious from this point of view.

### 3.4. Comparison of the Structures of Aptamer–Thrombin Complexes

If aptamer activity has no direct relationship with its thermodynamic characteristics, it could be assumed that bimodular aptamers have more favorable contacts within the complex with thrombin. Structures of NU172–thrombin complexes with two different coordinated cations have been published recently; however, their resolution is 2.5–2.8 Å [20]. One more complex of bimodular aptamer RE31 with thrombin has a resolution of 2.98 Å. Comparison of number of polar contacts, interface area and number of atoms in the interface for HD1, NU172 (Na^+^), NU172 (K^+^), and RE31 complexes with thrombin gave no obvious correlation with aptamer affinity (Table 4).

**Figure 5:**
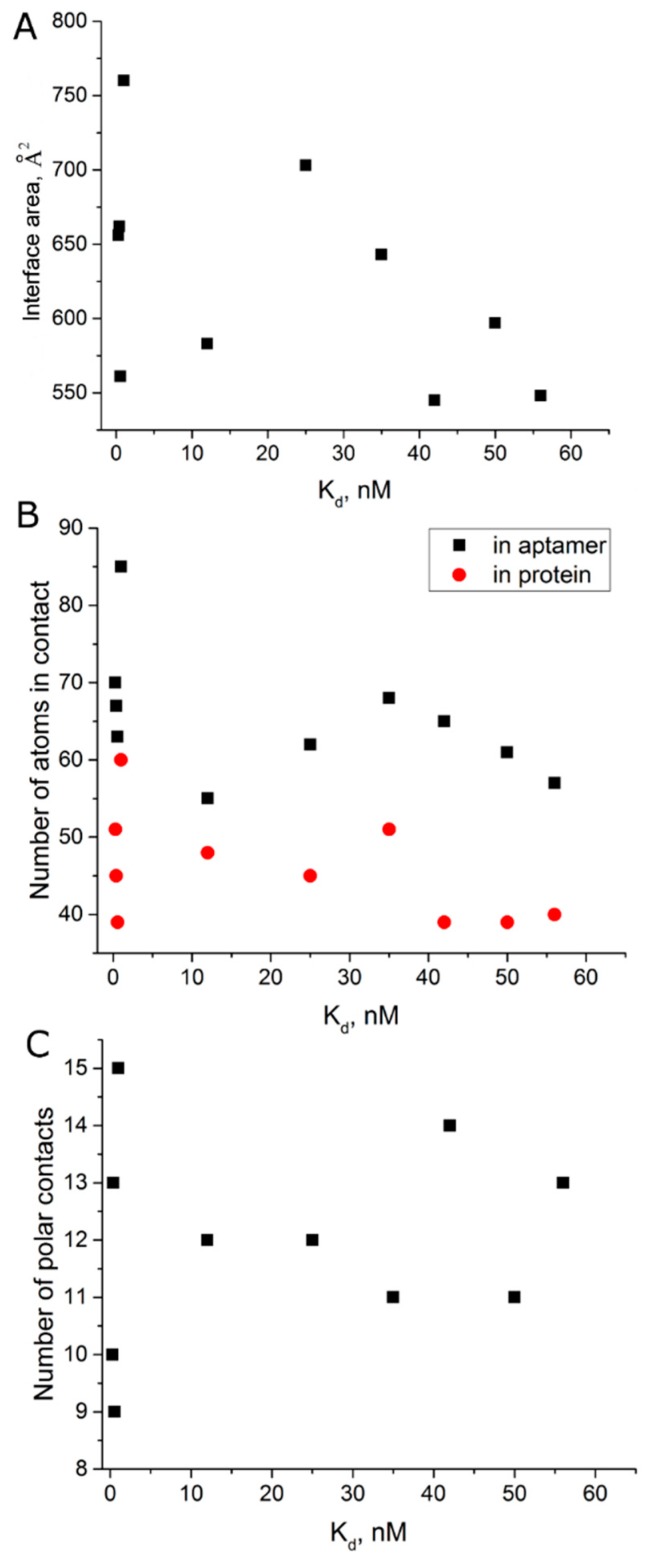
Analysis of gross structural parameters of aptamer–thrombin complexes revealed no correlation with dissociation constants. (**A**) interface area, (**B**) number of contacting atoms in contact in aptamer (black) and in protein (red), (**C**) number of polar contacts.

We performed an analysis of all known complexes of thrombin with HD1-like aptamers, that is the aptamers that bind to exosite I of thrombin (Table 4), in an attempt to reveal some general trends. Gross parameters such as interface area, number of polar contacts and number of contacting atoms were estimated. There is no strict correlation between the structural parameters of the complex and apparent dissociation constants (Figure 5).

In protein science, examples of high-affinity complexes with ‘hot spots’ are known [27]. ‘Hot spots’ can be represented by 1–2 hydrogen bonds that are responsible for a 10-fold decrease of the apparent dissociation constant. We performed a detailed analysis of polar contacts in complexes of thrombin with aptamers HD1, T4K (with 3-acetyl-3-amino1-propenyl instead of methyl in T4), and ΔT12 (apyrimidine site in T12) (Supplementary Table S5). The last two complexes have a 100-fold difference in apparent dissociation constants; therefore, these complexes are expected to have evident differences within their interfaces. However, nearly all polar contacts were the same. The small differences in distances and angles could not result in the drastic change of affinity. It is notable that the interface of the thrombin–RE31 complex has formally the smallest amount of polar contacts.

Interface area is an indirect estimation of hydrophobic effect during complex formation. Recently, it was shown that aptamers with modified nucleotides show correlation between interface area and logarithm of apparent association constant for complex formation, lnK_a_ [8]. However, there is no that kind of correlation for thrombin aptamers. Thus, the affinity of thrombin aptamers could not be explained by the gross structural features of the final complex. Some other aspects exist that are crucial for establishing a high affinity.

## 4. Discussion

G-quadruplex aptamers complexed to thrombin exosite I have the topology of antiparallel G-quadruplexes with two G-quartets, which are stabilized by K^+^ in the center and covered with loops on the top and on the bottom [28,29]. A set of homologous bimodular aptamers are known to have 30–40-fold lower dissociation constants for complexes with thrombin than the simplest 15-meric G-quadruplex aptamer, HD1 [9,10,12]. It has been shown for the NU172–thrombin and RE31–thrombin complexes that the duplex module stacks with hinge loops, those, in turn, are stacked with the lateral three-nucleotide loop and G-quadruplex [19,20]. However, it is not obvious why the affinity rises, as the conformation of G-quartets and recognizing loops as well as interface organization in the complex are very similar to those for the HD1–thrombin complex. Several reasons could be responsible for this phenomenon; the most obvious ones are discussed below.

### 4.1. Conformational Polymorphism as a Reason for Reduced Functional Activity

The conformation of the G-quadruplex module of thrombin aptamers is rather dynamic; the salt composition of the buffer significantly affects both aptamer conformation and functional activity [13]. Thus, strict comparison of different aptamers requires uniform conditions. Unlike the majority of previous experimental studies, which utilize saturating concentrations of K^+^ cations, here we used buffer with a physiological level of relevant cations similar to blood plasma: 140 mM NaCl and 10 mM KCl. These conditions are more appropriate for studying structure–functional aspects of the aptamers as potential inhibitors of thrombin activity, although the stability of the G-quadruplex module is decreased significantly. For example, the melting temperature of the NU172 G-quadruplex is decreased by 10 °C (Table 2) in physiological buffer with 10 mM KCl, compared to the temperature for the sample in buffer with 110 mM KCl [30]. An advantage of this condition is that it does not stiffen the aptamer structure and allows the dynamic behavior of the aptamer to be followed.

Only three of the eight bimodular aptamers, jjj (31-TBA), aaa (NU172), and aaj, adopted uniform conformations of the monomolecular G-quadruplex that is in equilibrium with the unfolded state, a melting globe (Figure 2, Figure 3 and Figure S1). The duplex module is more stable than the G-quadruplex one; under the conditions used, melting of these bimodular aptamers could be described as a two-step process involving transition from the bimodular structure to a hairpin and subsequent unfolding of the hairpin

$$G_{f}-D_{f}\underset{\leftarrow }{\to }G_{u}-D_{f}\underset{\leftarrow }{\to }G_{u}-D_{u}$$

where ‘G’ is G-quadruplex module, ‘D’ is duplex module, ‘f’ and ‘u’ refer to folded and unfolded states, respectively.

One aptamer, jja, adopted a conformation of antiparallel G-quadruplex that is in equilibrium with a dimer with parallel G-quadruplex topology (Figure 2 and Figure 3). Two of the eight bimodular aptamers, jaa and jaj, are monomolecular antiparallel G-quadruplexes, the unfolding of which proceeds through several intermediates with non-defined G-quadruplex topology (Figure 2, Figure 4 and Figure S1). The last two aptamers, aja and ajj, are a mix of several oligomeric forms, namely, dimers and tetramers with parallel G-quadruplex topology (Figure 2, Figure 4 and Figure S1).

Aptamers with formally identical proposed bimodular structures have different conformational landscapes that affect the affinity/inhibitory constants. For dimeric hybrid aptamers aja and ajj, exchanging the modules yielded blocks of 6Gs, which is known to be favorable for highly stable parallel G-quadruplexes [31,32]. Hybrid aptamer jja has blocks with less Gs, that is, 3 and 4. It undergoes a unique two-state transition from monomolecular ‘antiparallel’ to dimeric or tetrameric ‘parallel’ G-quadruplex. Here, the functional activity is completely in agreement with the amount of monomolecular species: it is drastically decreased in the order jja (71%) > aja (26%) > ajj (16%) and the monomolecular fraction yielded apparent inhibitory constants of 1.2, 13.2 and 48.6 nM [9], respectively.

In general, the duplex module could serve as a ‘restrictor’ of the amount of possible conformations, as illustrated by comparison of NU172 and its G-quadruplex module, NU, alone (Figure 3). However, this is not true for 31-TBA and its G-quadruplex module, HD1, because of the intrinsic conformational homogeneity of HD1 (Figure 3).

**Figure 6:**
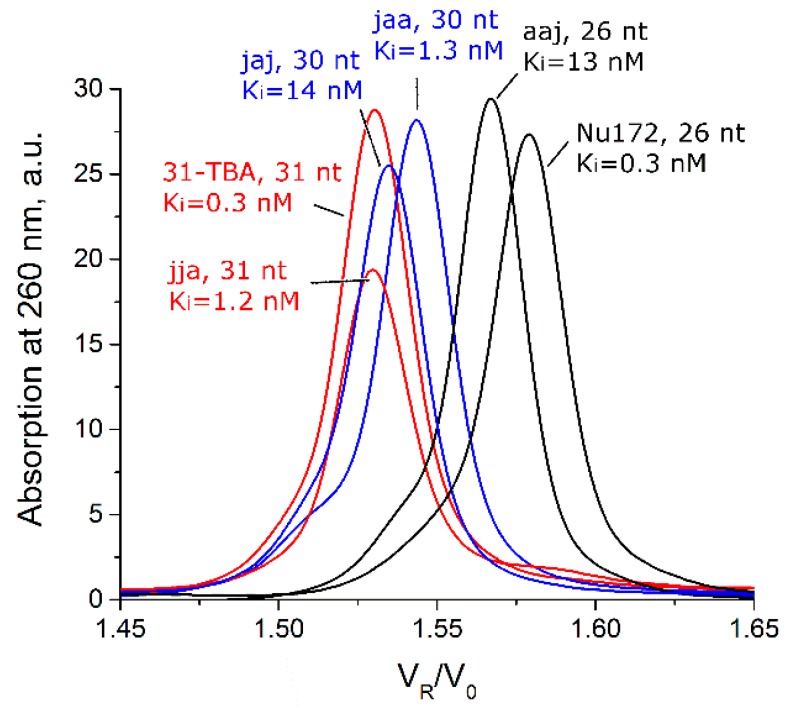
Comparison of different monomeric forms of bimodular aptamers in size-exclusion high performance liquid chromatography. Aptamers with equal lengths are colored identically. V_R_/V_0_ is relative retention volume. Ki: is apparent inhibitory constant from [9].

It is clear that there are more successful combinations of two different modules, which provide high inhibitory activity, K_i_ < 1.5 nM, as well as less successful combinations with K_i_ > 13 nM. What is the reason for the 10-fold gap in the inhibitory constants? We compared the size-exclusion chromatograms of monomeric fractions of different bimodular aptamers and found clear differences between aptamers with high and low levels of inhibitory activity (Figure 6). Comparing aptamers with the same lengths and molecular weights (deviations are less than 0.4%), we can conclude that highly active aptamers are more compact/structured. Their counterparts with low activity, jaj and aaj, have decreased melting temperatures (32–34 °C), which are even lower than that for G-quadruplex module, HD1 (39 °C; Table 2). A possible reason for this destabilization effect could be explained by insufficient stacking between G-quadruplex, duplex modules and hinge loops. Further structural studies are required to find out the reasons of the effect.

### 4.2. Thermodynamic Aspects of Bimodular Aptamers

Thermodynamic parameters for unfolding of the G-quadruplex module were estimated for aptamers with single transitions, jjj (31-TBA), aaa (NU172), jja, aaj, and 00j (HD1), according to Mergny and Lacroix [16] (Table 3, Figures S2 and S3). Unfolding of G-quadruplex modules of 31-TBA and HD1 had the highest change of enthalpy and entropy. The values for 31-TBA and HD1 are very similar, indicating similarity in G-quadruplex organization in these two aptamers. Aptamers aaa (NU172) and jja have the same sequence of G-quadruplex module, but different transition types; nevertheless, their thermodynamic characteristics are also very similar. The least stable G-quadruplex of aaj (melting temperature of only 32 °C) had the smallest changes of enthalpy and entropy. The structure of the junction hinge between two modules is responsible for tiny differences in the stability of bimodular aptamers; the amount of purines in the lateral 3-nucleotide loop and hinge loops correlates with the increased thermal stability of the G-quadruplex module.

The thermodynamic characteristics of G-quadruplexes of 31-TBA and HD1 are very similar, but their functional activity varies 40-fold. This formal comparison of G-quadruplex status again yielded no clue to the structure–affinity relationship.

### 4.3. Possible Mechanism for Complex Formation between Aptamer and Thrombin

Comparing the structures of HD1-like aptamer–thrombin complexes has yielded no obvious structure–activity relationships. Apparent equilibrium dissociation constants did not correlate with parameters of complexes, like interface area, number of polar contacts or number of contacting atoms (Figure 5). Moreover, aptamers T4K and ΔT12 with 100-fold difference in affinity have identical sets of polar contacts in the complex with thrombin. Thus, the structures of the final complexes of different HD1-like aptamers with thrombin are very similar, and parameters of the complexes do not specify the affinity of the aptamers. As far as mono- and bimodular aptamers are concerned, the initial and final states of macromolecules in complex formation are almost equal for different aptamers, and large differences in affinity are provided as a result of peculiarities of association process.

Kinetic constants were determined for thrombin complex formation with RE31 and HD1 [12,33]. The apparent association kinetic constant for bimodular RE31 is 55-fold higher than that for monomodular HD1, whereas the apparent dissociation kinetic constants differ by only two-fold. Thus, during the association process bimodular aptamers have significant benefit over the single G-quadruplex module. One more essential point is the comparable values of enthalpy and entropy changes upon HD1 folding and formation of the thrombin complex. The values for HD1–thrombin complex formation are ΔH° = −89–110 kJ/mol and ΔS° = −149–240 J/mol [25,34], whereas values for HD1 folding are ΔH° = −96–160 kJ/mol and ΔS° = −300–490 J/mol [25,30,34].

**Figure 7:**
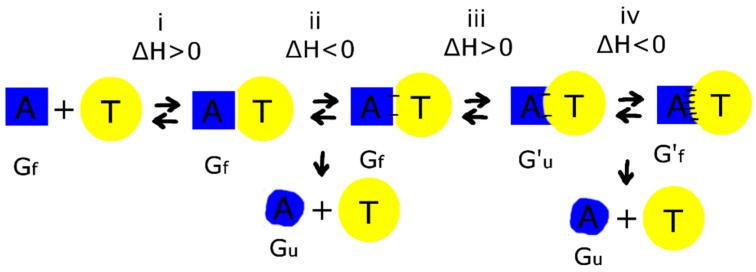
Schematic representation of the stages of complex formation between aptamer (A) and thrombin (T). Black dashes are hydrogen bonds. G and G′ are two different conformations of the G-quadruplex module, ‘f’ and ‘u’ refer to folded and unfolded states, respectively. Enthalpy favorable stages yield ‘side product’—an unfolded G-quadruplex module that dissociates from the intermediate complex.

Therefore, a tentative mechanism of complex formation could be: (i) the aptamer approaches thrombin, and water is excluded from the interface (ΔH° > 0); (ii) the first polar contacts are formed (ΔH° < 0), the energy is redistributed between macromolecule and the milieu; (iii) energy-consuming conformational rearrangement of the aptamer happens, namely the TT pair breaks, and recognizing loops adopt favorable conformation for the interaction with thrombin (ΔH° > 0); (iv) the formation of the complex is completed with formation of additional polar contacts (ΔH° < 0); the energy is redistributed between macromolecule and the milieu (Figure 7).

As far as the overall enthalpy of the binding process is comparable to the enthalpy of aptamer unfolding, enthalpy of binding could yield partial disordering of the aptamer structure. Consequently, premature dissociation of the disordered aptamer before the final complex formation is happened. High-affinity bimodular aptamers, like 31-TBA, have the same thermodynamic parameters as the G-quadruplex alone, HD1. The duplex module has higher stability than the G-quadruplex one; therefore, after G-quadruplex melting the 31-TBA become a hairpin, contrary to HD1 being completely melt. The rate of G-quadruplex refolding is probably higher in the case of 31-TBA due to fixation of the ends by the hairpin. As described above, during complex formation disordered HD1 dissociates from the thrombin surface before the final complex formation. On the contrary, bimodular aptamers are able to manage refolding of the G-quadruplex module before dissociation. This suggestion could explain the high apparent kinetic constant of association and, therefore, high affinity of bimodular aptamers compared to the G-quadruplex alone. Further studies of the kinetic aspects of aptamer–thrombin complexes will be of high value for the fundamental understanding details of recognition events.

## 5. Conclusions

This research is focused on estimating a thermodynamic background and the conformational heterogeneity of thrombin DNA aptamers with G-quadruplex and duplex modules. Spectroscopic and chromatographic techniques revealed the conformational homogeneity of aptamers with high inhibitory activity, as well as a mixture of monomeric and oligomeric species for aptamers with low inhibitory activity. Thermodynamic parameters for aptamer unfolding were calculated, and their correlation with aptamer functional activity was found. Detailed analysis of HD1-like aptamer-thrombin complexes revealed the similarity of the interfaces of aptamers with drastically different affinities to thrombin. Putative mechanism was proposed based on the supposition of kinetic events during complex formation that have a larger impact on the affinity than the states of initial and final macromolecules. Duplex module stabilizes G-quadruplex core during association phase preventing partial denaturation of G-quadruplex module.

## Figures and Tables

**Table 1:** Oligomeric composition of bimodular aptamers estimated by size-exclusion chromatography.

Aptamer	Monomeric Form			Dimeric Form			Tetrameric Form		
	*V*_R_/ *V*_0_	*M*_calculated_/ *M*_monomer_	Quantity (%)	*V*_R_/*V*_0_	*M*_calculated_/ *M*_monomer_	Quantity (%)	*V*_R_/*V*_0_	*M*_calculated_/ *M*_monomer_	Quantity (%)
31-TBA (jjj)	1.53	0.96	100	-	-	-	-	-	-
jaa	1.54	0.91	100	-	-	-	-	-	-
jja	1.53	0.93	71	1.24	2.81	29	-	-	-
NU172 (aaa)	1.58	0.92	100	-	-	-	-	-	-
aaj	1.57	0.97	100	-	-	-	-	-	-
jaj	1.53	0.95	100	-	-	-	-	-	-
aja	1.57	0.91	26	1.29	2.65	59	1.12	5.16	14
ajj	1.56	0.95	16	1.29	2.67	41	1.12	5.29	42

V_R_/V_0_ = relative retention volume, M_calculated_/M_monomer_ = molecularity. The aptamers are grouped by their functional activity: aK_i_ < 1.5 nM, aK_i_ ≈ 14 nM and aK_i_ ≈ 50 nM.

**Table 2:** Properties of the set of aptamers obtained from CD and UV melting experiments.

Aptamer	G-Pattern	G-Quadruplex Topology	Transition	Melting Temperature ± SD, °C		*aK*_i_ ± SD, nM [9,10]
				G-Quadruplex Module	Duplex Module	
31-TBA (jjj)	2-2-2-2-4	A	A → U	42.7 ± 1.0	54.4 ± 1.0	0.34 ± 0.10
jaa	2-2-3-3-2	A	A → U + P	41 ± 4	54.2 ± 0.8	1.34 ± 0.05
jja	2-2-3-3-4	A	A → P	49 ± 2	58.9 ± 1.5	1.2 ± 0.3
NU172 (aaa)	2-3-3-2-2	A	A → U	37.1 ± 1.0	57 ± 3	0.29 ± 0.06
HD1 (00j)	2-2-2-2	A	A → U	39.2 ± 0.8	-	14.7 ± 1.0
aaj	2-2-2-2-2	A	A → U	32 ± 3	58 ± 3	13.4 ± 1.8
jaj	2-2-2-2-2	A	A → U + P	34.4 ± 1.0	50.8 ± 1.2	14.2 ± 0.7
aja	2-3-3-6	P + A	A → ?	49.3 ± 0.8	n.d.	13.2 ± 1.6
ajj	2-2-2-6	P	P → P	47 ± 2	n.d.	48.6 ± 0.2
NU (00a)	2-3-3-2	A	A → U + P	17 ± 2	-	46 ± 3

‘G-pattern’ numbers show the lengths of G-blocks in the aptamer sequence. The topologies of the G-quadruplexes were determined from the characteristic maxima in the CD spectra: A = antiparallel, P = parallel, U = unfolded; transitions during thermal melting are listed. Colored lines correspond to the aptamers with two-state melting processes. The aptamers are grouped by their functional activity: aK_i_ < 1.5 nM, aK_i_ ≈ 14 nM and aK_i_ ≈ 50 nM. SD is standard deviation. n.d. = not determinable.

**Table 3:** Thermodynamic parameters of G-quadruplex modules of the aptamers with a single transition during thermal melting.

Aptamer	Transition	Δ*H*^o^, kJ/mol	Δ*S*^o^, J/mol	Melting Temperature, °C			*aK*_i_, nM [9]
				G-Quadruplex Module	Δ*T*	Duplex Module	
31-TBA (jjj)	A → U	−136 ± 12	−430 ± 40	42.7 ± 1.0	+3.5	54.4 ± 1.0	0.34 ± 0.10
NU172 (aaa)	A → U	−122 ± 3	−393 ± 11	37.1 ± 1.0	−2.1	57 ± 3	0.29 ± 0.06
jja	A → P	−121 ± 11	−380 ± 30	49 ± 2	+9.8	58.9 ± 1.5	1.2 ± 0.3
HD1 (00j)	A → U	−138 ± 3	−443 ±10	39.2 ± 0.8	0	-	14.7 ± 1.0
aaj	A → U	−107 ± 4	−347 ± 13	32 ± 3	−7.2	58 ± 3	13.4 ± 1.8

The data were calculated from CD melting curves (295 nm) by using a ‘two-state process’ assumption. SD were calculated. Melting temperatures and inhibitory constants are shown for comparison. ΔT values correspond to changes in G-quadruplex melting temperature relatively HD1 aptamer.

**Table 4:** Characteristics of aptamer–thrombin complexes. The complexes are ordered according to their apparent dissociation constants.

Aptamer	PDB ID	*K*_d_, nM	Interface Area, Å^2^ [21]	Number of Polar Contacts	Number of Atoms in 4Å Proximity	
					Aptamer	Protein
ΔT3	4lz4 [22]	56 [23]	548	13	57	40
HD1 (Na^+^)	4dih [24]	50 [10]	597	11	61	39
ΔT12	4lz1 [22]	42 [23]	545	14	65	39
NU172 (Na^+^)	6gn7 [20]	35 [13]	643	11	68	51
mTBA	3qlp [21]	25 [25]	703	12	62	45
HD1	4dii [24]	12 [10]	583	12	55	48
T4W	6eo6 [26]	1 [26]	760	15	85	60
RE31	5cmx [19]	0.56 [12]	561	9	63	39
NU172	6evv [20]	0.29 [9]	656	10	70	51
T4K	6eo7 [26]	0.39 [26]	662	13	67	45

PDB ID is accession number of the structure in protein database (www.rcsb.org); K_d_ is apparent dissociation constant of thrombin-aptamer complex.

## Supplementary Materials

The following are available online at http://www.mdpi.com/2218-273X/9/2/41/s1; Table S1: Description of aptamers studied, Figure S1: Chromatograms of SEC HPLC; Table S2: Oligomeric composition of aptamer HD1, complementary oligonucleotide and their duplex estimated in SEC experiments; Table S3: Melting temperatures of aptamer structures derived from CD spectra; Table S4: Melting temperatures of aptamer structures derived from UV spectra; Figures S2 and S3: Melting curves derived from CD spectra and their linearization in coordinates lnK (1/T); Figures S4 and S5: Denaturation-renaturation experiments for G-quadruplex and duplex modules: 31-TBA, NU172, jja, aaj, and HD1; Table S5: Detailed analysis of polar contacts in the interface of complexes of thrombin and aptamers.
