# Supplementary material for: Putative Mechanisms Underlying High Inhibitory Activities of Bimodular DNA Aptamers to Thrombin
Source: Biomolecules. 2019 Jan 24;9(2):41. doi: 10.3390/biom9020041 (PMC6406280; doi:10.3390/biom9020041)
Supplement: Supplementary file 1 [file biomolecules-09-00041-s001.pdf]

Putative Mechanisms Underlying High Inhibitory Activities of Bimodular DNA Aptamers to Thrombin

Elena G. Zavyalova[1,*], Valeria A. Legatova[1], Rugiya Sh. Alieva[1], Artur O. Zalevsky[2], Vadim N. Tashlitsky[1], Alexander M. Arutyunyan[3], Alexey M. Kopylov[1]

[1] Chemistry Department, Lomonosov Moscow State University, Moscow, 119991, Russian Federation
[2] Department of Bioinformatics and Bioengineering, Lomonosov Moscow State University, Moscow, 119991, Russian Federation
[3] Belozersky Research Institute of Physico-Chemical Biology, Lomonosov Moscow State University, Moscow, 119991, Russian Federation

* To whom correspondence should be addressed. Tel: +74959393149; Fax: +74959393181; Email: zlenka2006@gmail.com

Present Address: Elena Zavyalova, Chemistry Department, Lomonosov Moscow State University, Moscow, 119991, Russian Federation

Supplementary materials

Table S1. Description of aptamers studied: sequence, length, molecular weight and inhibitory constants determined earlier. 31-TBA-related fragments are shown in lower case, NU172-related fragment are shown in upper case. A three-letter code for chimeric aptamers has been designed to indicate the origin of corresponding modules in the following order: duplex - hinge loops - G-quadruplex, where 'j' corresponds to 31-TBA and 'a' corresponds to NU172.

Aptamer	Sequence	Length, nucleotides	Molecular weight	aK_i±SD, nM [1,2]
31-TBA (jjj)	cactgg ta ggttggtgtggttgg gg ccagtg	31	9710	0.34±0.10
jaa	cactgg TA GGTTGGGTAGGGTGG T ccagtg	30	9390	1.34±0.05
jja	cactgg ta GGTTGGGTAGGGTGG gg ccagtg	31	9744	1.2±0.3
ajj	CGCC ta ggttggtgtggttgg gg GGCG	27	8476	48.6±0.2
aaj	CGCC TA ggttggtgtggttgg T GGCG	26	8121	13.4±1.8
aja	CGCC ta GGTTGGGTAGGGTGG gg GGCG	27	8510	13.2±1.6
jaj	cactgg TA ggttggtgtggttgg T ccagtg	30	9356	14.2±0.7
NU172 (aaa)	CGCC TA GGTTGGGTAGGGTGG T GGCG	26	8155	0.29±0.06
HD1 (00j)	ggttggtgtggttgg	15	4726	14.7±1.0
NU (00a)	GGTTGGGTAGGGTGG	15	4760	46±3

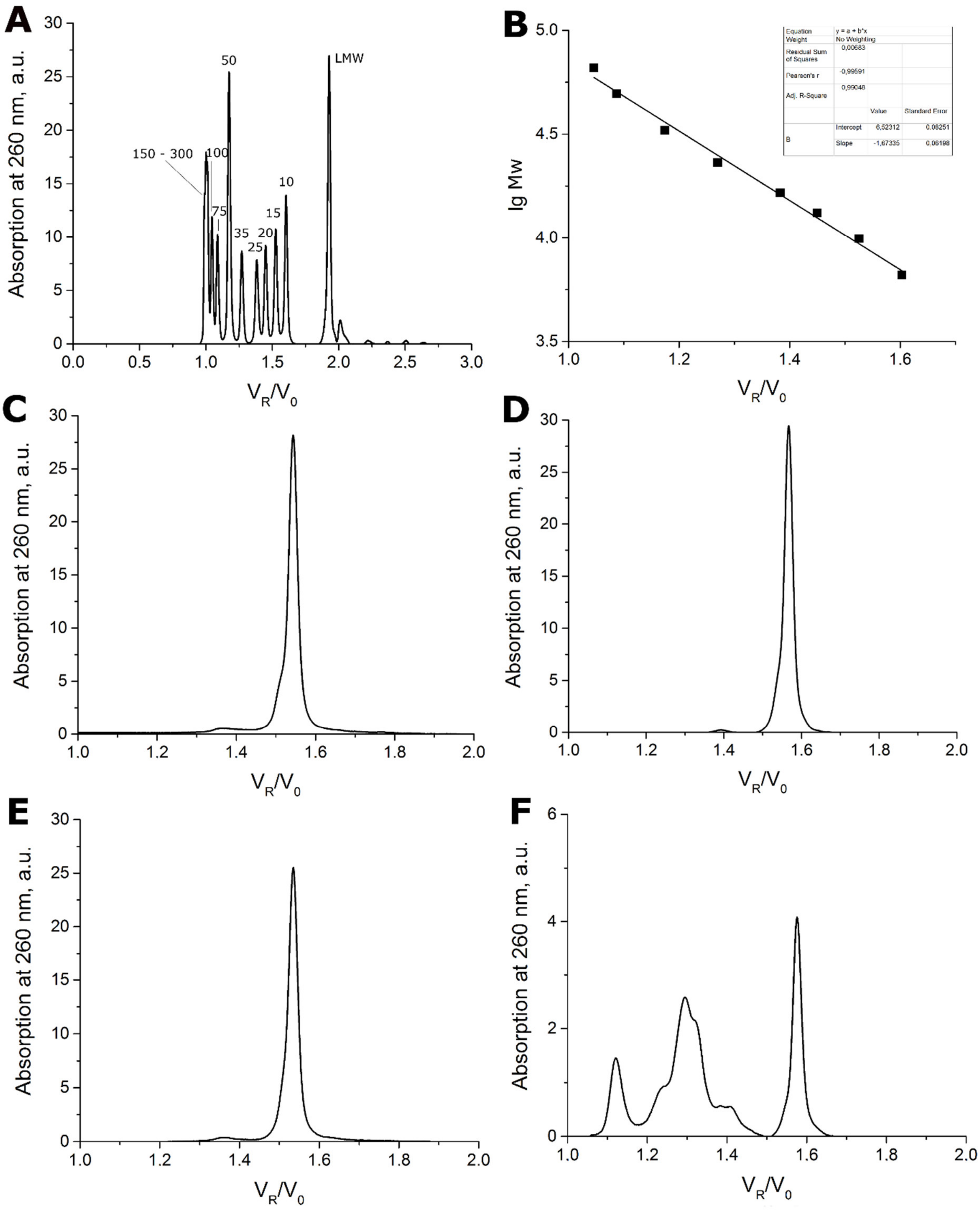

Figure S1. Chromatograms of SEC HPLC for DNA ladder (A), calibration curve and its linearization with R^2=0.99 (B), aptamers jaa (C), aaj (D), jaj (E) and aja (F).

Table S2. Oligomeric composition of aptamer HD1, complementary oligonucleotide and their duplex estimated in SEC experiments. V_R/V_0 - relative retention volume, $M_{calculated}/M_{monomer}$ – molecularity.

	V_R/V_0	$M_{calculated}/M_{monomer}$	Quantity, %
HD1, ggttggtgtggttgg	1.65	1.19	100
cHD1, ccaaccacaccaacc	1.62	1.46	100
HD1+cHD1	1.52	1.02	100

Table S3. Melting temperatures of aptamer structures derived from CD spectra at different wavelengths. A – antiparallel G-quadruplex, P – parallel G-quadruplex, U – unfolded G-quadruplex. Standard deviations are provided.

Aptamer	Melting temperatures, ºC				Average value	Transition
	295 nm	267 nm	247 nm	260 nm		
31-TBA=jjj	43.4±0.5	42.2±0.7	42.6±1.0	n.d.	42.7±1.0	A→U
jaa	38.0±0.6	23±4	43.2±1.5	n.d.	41±4	A→U+P
jja	47.8±0.7	47.7±1.9	51.8±0.7	47.2±1.8	49±2	A→P
ajj	45.2±1.6	n.d.	n.d.	48.0±0.5	47±2	P→P
aaj	35.3±0.3	30.4±0.8	31.2±1.6	n.d.	32±3	A→U
aja	49.3±0.8	n.d.	n.d.	n.d.	49.3±0.8	A→?
jaj	33.8±0.6	25.9±1.3	34.9±1.9	n.d.	34.4±1.0	A→U+P
NU172=aaa	37.2±0.4	36.3±1.0	37.7±0.6	n.d.	37.1±1.0	A→U
HD1=00j	38.8±0.2	40.3±0.3	38.4±0.2	-	39.2±0.8	A→U
NU=00a	17±2	n.d.	n.d.	-	17±2	A→U+P

Table S4. Melting temperatures of aptamer structures derived from UV spectra at different wavelengths. The values from CD and UV (295 nm) are rather similar, as both characterize melting of G-quadruplex. The values from UV (260 nm) are 10ºC higher than those for G-quadruplex elements that illustrates duplex stability under conditions used. A – antiparallel G-quadruplex, P – parallel G-quadruplex, U – unfolded G-quadruplex. Standard deviations are provided.

Aptamer	Transition	Melting temperatures, ºC		
		G-quadruplex module		Duplex module
		Average value from CD	UV at 295 nm	UV at 260 nm
31-TBA jjj	A→U	42.7±1.0	45.7±0.5	54.4±1.0
jaa	A→U+P	41±4	45.3±0.7	54.2±0.8
jja	A→P	49±2	49.9±1.5	58.9±1.5
ajj	P→P	47±2	n.d.	47.8±0.8
aaj	A→U	32±3	33.8±1.0	58±3
aja	A→?	49.3±0.8	n.d.	n.d.
jaj	A→U+P	34.4±1.0	n.d.	50.8±1.2
NU172 aaa	A→U	37.1±1.0	40±2	57±3
HD1=00j	A→U	39.2±0.8	38.0±1.0	-
NU=00a	A→U+P	17±2	n.d.	-

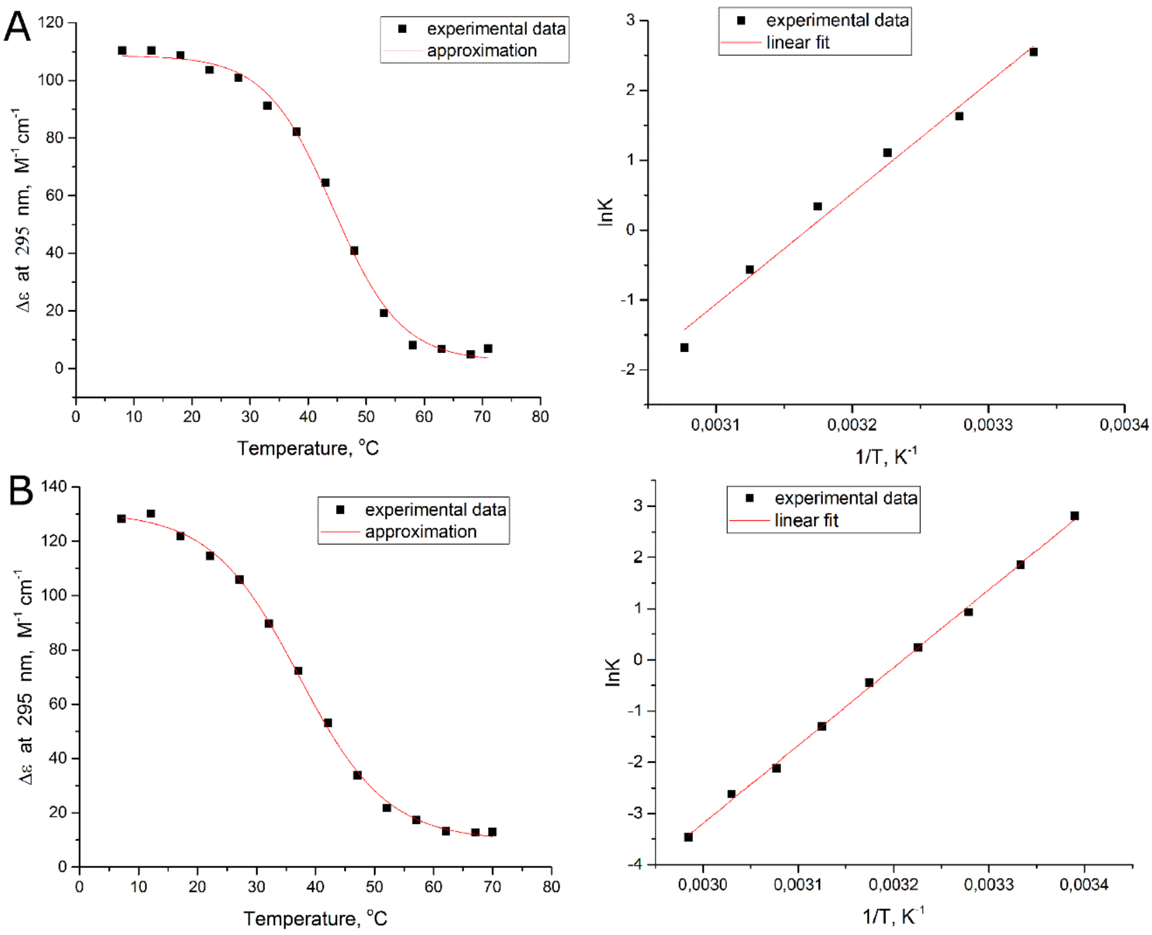

Figure S2. Melting curves derived from CD spectra and their linearization in coordinates lnK (1/T) for aptamers 31-TBA (A) and NU172 (B).

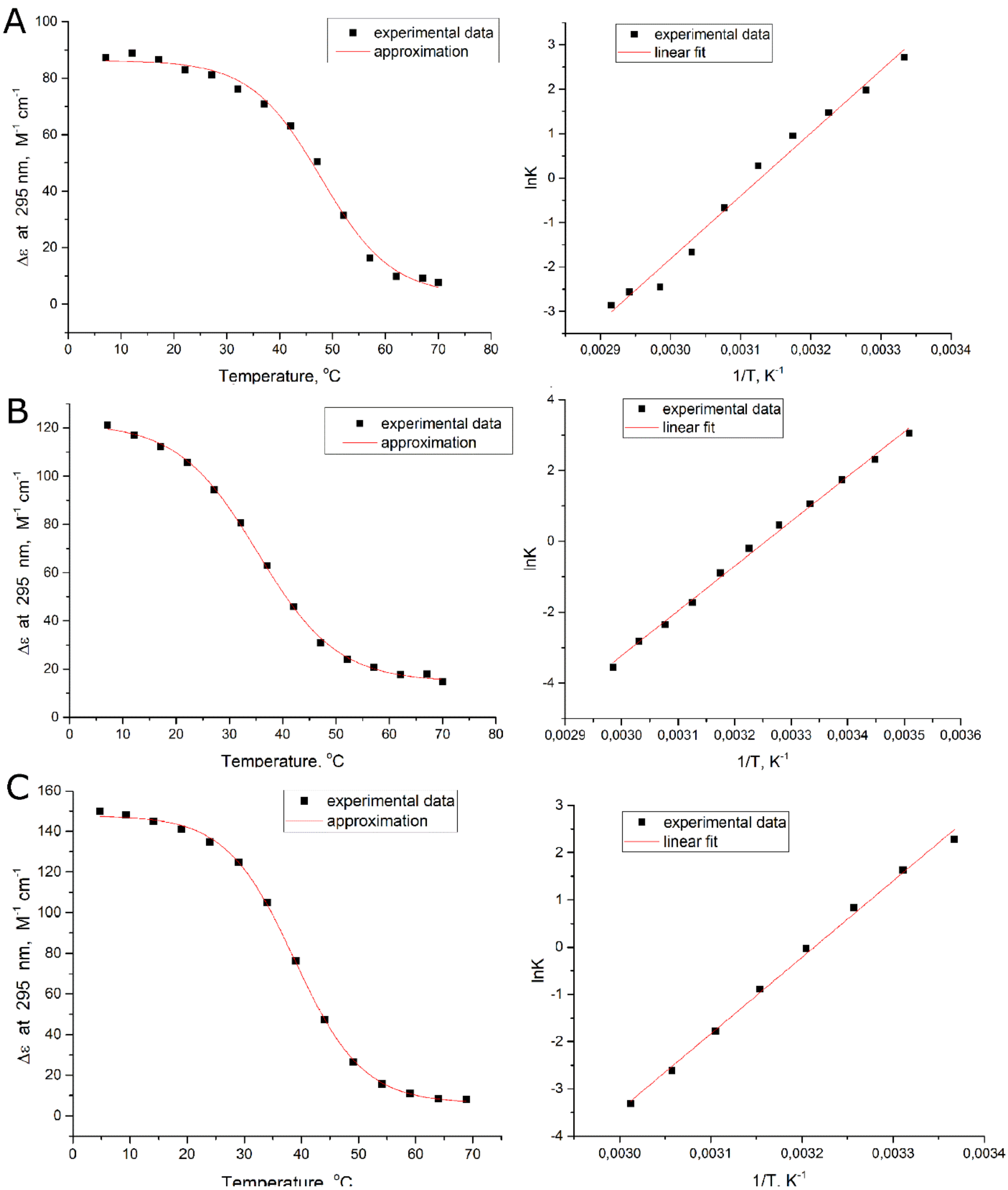

Figure S3. Melting curves derived from CD spectra and their linearization in coordinates lnK (1/T) for aptamers jja (A), aaj (B) and HD1 (C).

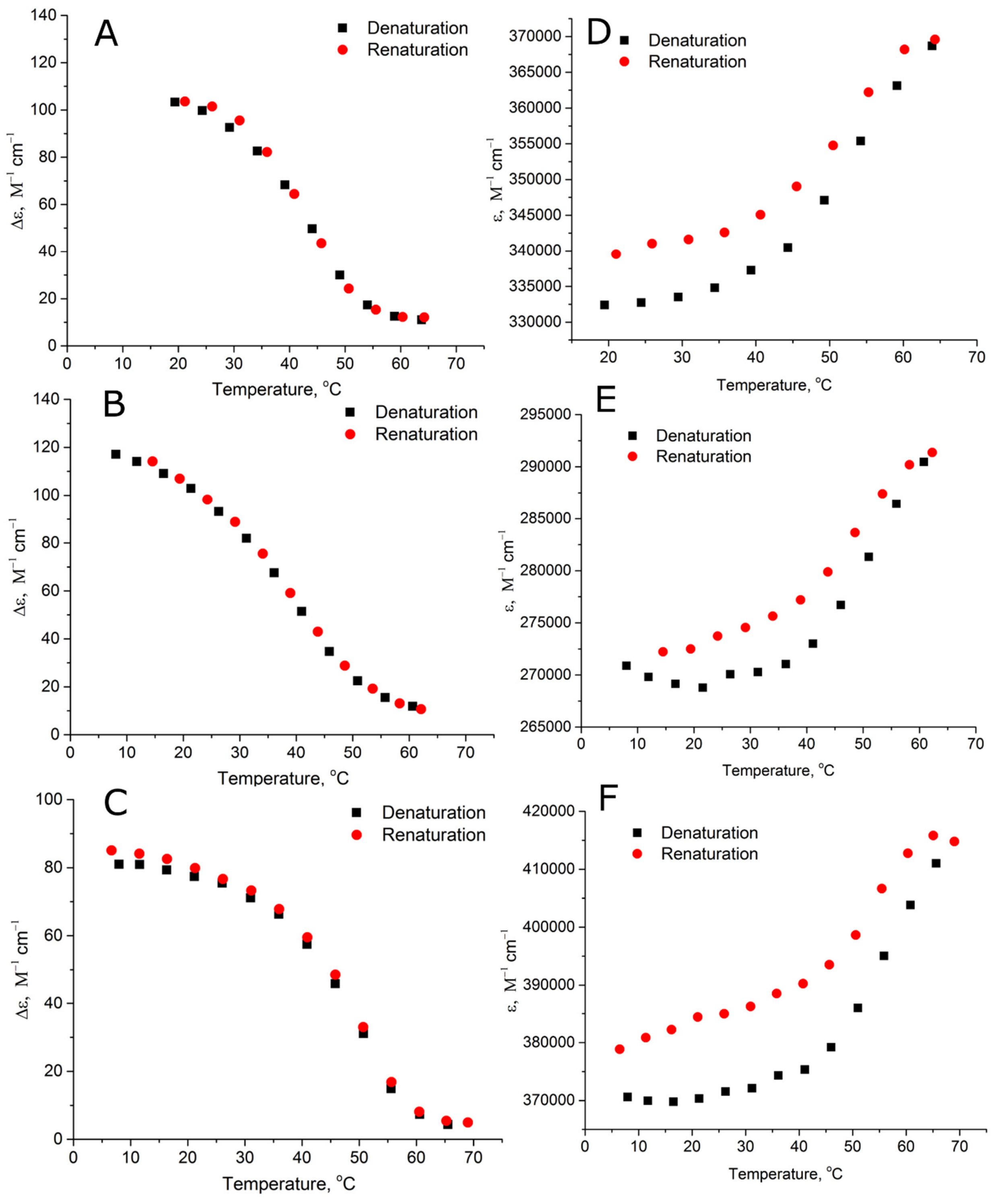

Figure S4. Denaturation-renaturation experiments for G-quadruplex (CD: A, B, C) and duplex (UV: D, E, F) modules: 31-TBA (A, D), NU172 (B, E), and jja (C, F).

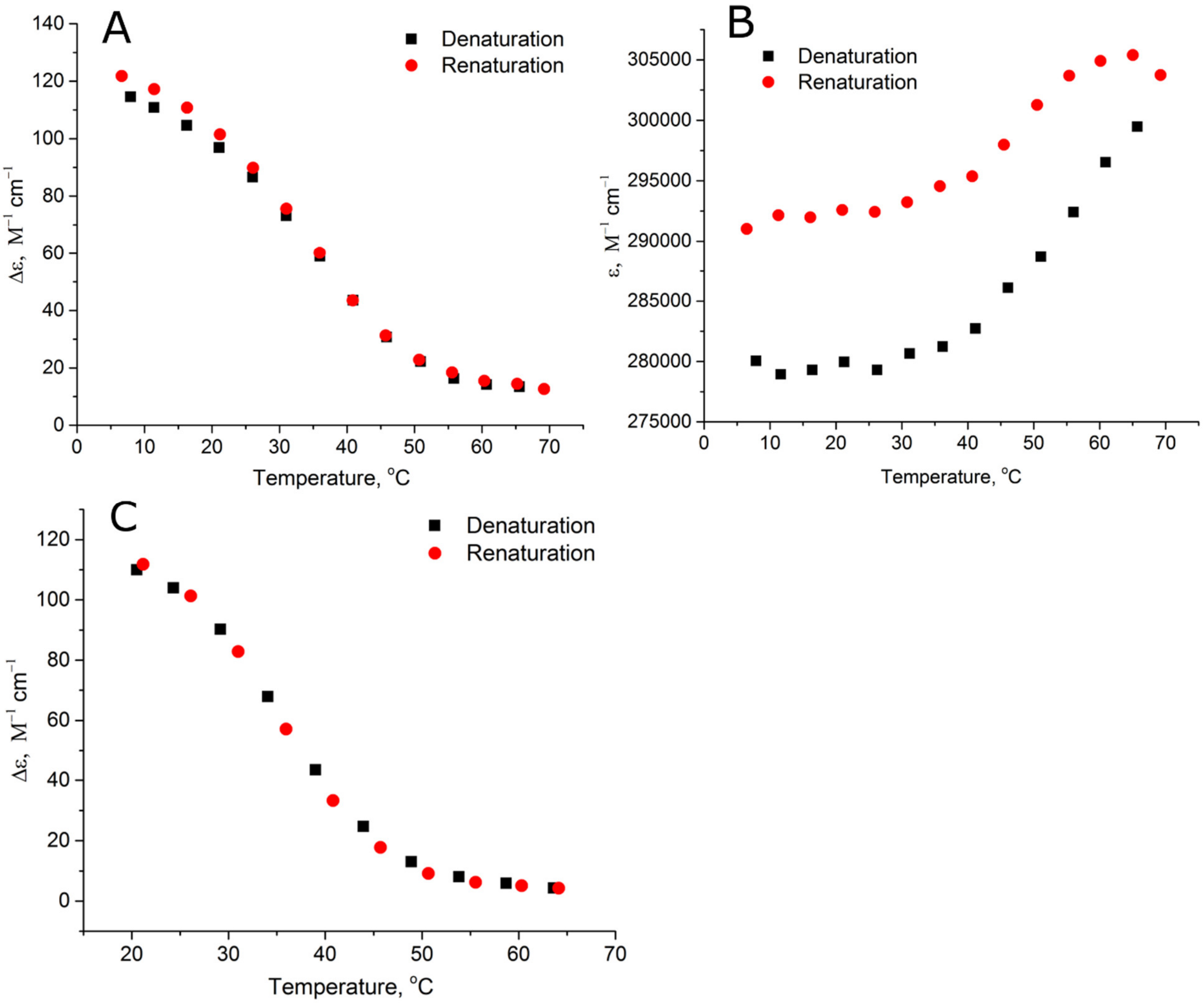

Figure S5. Denaturation-renaturation experiments for G-quadruplex (CD: A, C) and duplex (UV: B) modules: aaj (A, B) and HD1 (C).

Table S5. Detailed analysis of polar contacts in the interface of complexes of thrombin and aptamers HD1 (4dii), T4K (6eo7) and ΔT12 (4lz1). Identical contacts are shown in the same line.

4dii					4lz1					6eo7				
	Atom from aptamer	Atom from thrombin	Dis-tance, Å	Angle, º		Atom from aptamer	Atom from thrombin	Dis-tance, Å	Angle, º		Atom from aptamer	Atom from thrombin	Dis-tance, Å	Angle, º
1	O4' T13	Nα Y76	3.1	116.1	1	O4' T13	Nα Y76	2.9	108.0	1	O4' T4	Nα Y76	2.9	113.5
2	O2 T13	Nδ R75	3.2	100.5										
3	O2 T13	Nε R75	3.3	99.0	2	O2 T13	Nε R75	3.0	132.1	2	O2 T4	Nε R75	2.9	130.4
4	O4 T4	Nε R75	2.5	119.6	3	O4 T4	Nε R75	2.8	96.9	3	O4 T13	Nε R75	2.8	98.1
5	O4 T4	Nε' R75	3.3	79.2	4	O4 T4	Nε' R75	2.8	96.0	4	O4 T13	Nε' R75	2.8	94.7
					5	O4 G14	Nε R75	3.5	117.0					
					6	O4 T3	Nε' R75	3.3	149.0	5	O4 T12	Nε' R75	3.2	143.0
					7	N3 T3	Oδ E77	2.9	118.9	6	N3 T12	Oδ E77	2.8	117.1
6	O4' T3	O_{OH} Y117	2.8	117.9	8	O4' T3	O_{OH} Y117	2.9	122.4	7	O3' T12	O_{OH} Y117	3.2	121.1
7	O3' T4	Nγ N78	3.4	118.4	9	O3' T4	Nγ N78	3.6	116.1	8	O3' T13	Nγ N78	3.4	124.8
					10	O_{PO4} G5	Nγ N78	3.1	156.2	9	O_{PO4} G14	Nγ N78	3.1	158.5
8	O4' G5	Nε R77A	2.5	108.7	11	O4' G5	Nε R77A	3.1	97.6	10	O4' G14	Nε R77A	3.0	98.6
9	O2 T4	Nε' R77A	2.8	140.8	12	O2 T4	Nε' R77A	3.0	132.2	11	O2 T13	Nε' R77A	3.0	128.9
10	O5' G5	Nε' R77A	3.5	117.1	13	O5' G5	Nε' R77A	3.4	134.0	12	O5' G14	Nε' R77A	3.4	132.9
11	O4' G5	Nε' R77A	3.2	76.0	14	O4' G5	Nε' R77A	3.2	91.4	13	O4' G14	Nε' R77A	3.2	91.4

REFERENCES

1. Zavyalova,E., Golovin,A., Pavlova,G. and Kopylov,A. (2013) Module-activity relationship of G-quadruplex based DNA aptamers for human thrombin. *Curr. Med. Chem.*, **20**, 4836-4843.

2. Zavyalova,E. and Kopylov,A. (2016) G-quadruplexes and i-motifs as scaffolds for molecular engineering of DNA aptamers. In Santos, H. (ed.), *G-Quadruplex Structures, Formation and Roles in Biology*. Nova Publishers, New York, pp. 53–80.
